# Balancing multiple objectives in conformation sampling to control decoy diversity in template-free protein structure prediction

**DOI:** 10.1186/s12859-019-2794-5

**Published:** 2019-04-25

**Authors:** Ahmed Bin Zaman, Amarda Shehu

**Affiliations:** 1 Department of Computer Science, George Mason University, Fairfax, 22030 VA USA; 2 Department of Bioengineering, George Mason University, Fairfax, 22030 VA USA; 3 School of Systems Biology, George Mason University, Manassas, 20110 VA USA

**Keywords:** Protein energy landscape, Structural dynamics, Stochastic optimization

## Abstract

**Background:**

Computational approaches for the determination of biologically-active/native three-dimensional structures of proteins with novel sequences have to handle several challenges. The (conformation) space of possible three-dimensional spatial arrangements of the chain of amino acids that constitute a protein molecule is vast and high-dimensional. Exploration of the conformation spaces is performed in a sampling-based manner and is biased by the internal energy that sums atomic interactions. Even state-of-the-art energy functions that quantify such interactions are inherently inaccurate and associate with protein conformation spaces overly rugged energy surfaces riddled with artifact local minima. The response to these challenges in template-free protein structure prediction is to generate large numbers of low-energy conformations (also referred to as decoys) as a way of increasing the likelihood of having a diverse decoy dataset that covers a sufficient number of local minima possibly housing near-native conformations.

**Results:**

In this paper we pursue a complementary approach and propose to directly control the diversity of generated decoys. Inspired by hard optimization problems in high-dimensional and non-linear variable spaces, we propose that conformation sampling for decoy generation is more naturally framed as a multi-objective optimization problem. We demonstrate that mechanisms inherent to evolutionary search techniques facilitate such framing and allow balancing multiple objectives in protein conformation sampling. We showcase here an operationalization of this idea via a novel evolutionary algorithm that has high exploration capability and is also able to access lower-energy regions of the energy landscape of a given protein with similar or better proximity to the known native structure than several state-of-the-art decoy generation algorithms.

**Conclusions:**

The presented results constitute a promising research direction in improving decoy generation for template-free protein structure prediction with regards to balancing of multiple conflicting objectives under an optimization framework. Future work will consider additional optimization objectives and variants of improvement and selection operators to apportion a fixed computational budget. Of particular interest are directions of research that attenuate dependence on protein energy models.

## Background

Faster and cheaper high-throughput gene sequencing technologies have contributed millions of uncharacterized protein-encoding gene sequences in genomic databases [1]. Wet-laboratory efforts on resolving three-dimensional (tertiary) biologically-active/native structures of proteins have contributed an order of magnitude less [2]. This disparity and the recognition that tertiary structure determines to a large extent biological function and molecular mechanisms in the cell [3] motivate the development of complementary, computational approaches to tertiary protein structure prediction (PSP) [4].

Due to hardware and algorithmic improvements, template-free PSP methods, which focus on the most challenging setting of obtaining biologically-active structures of a protein from knowledge of its amino-acid sequence (in absence of a structural template from a close or remote homologous sequence), have made steady improvements in their capabilities [5]. Despite the success of hallmark protocols, such as Rosetta [6], Quark [7], and others [5], most notably due to domain-specific insight, template-free PSP presents outstanding computational challenges. The space of possible three-dimensional spatial arrangements of the chain of amino acids that constitute a protein molecule is vast and high-dimensional; we refer to this space as conformation space to recognize choices in the computational representation of a structure^1^. Exploration of such complex spaces is performed in a sampling-based manner (most commonly under the Metropolis Monte Carlo – MMC framework) and is biased by the internal energy that sums atomic interactions. The goal is to generate low-energy conformations that have a higher likelihood of being near-native conformations (and populating thermodynamically-stable regions of the energy surface) [8]. However, even state-of-the-art energy functions that quantify atomic interactions in a conformation are inherently inaccurate; they result in overly rugged energy surfaces (associated with protein conformation spaces) that are riddled with artifact local minima [9].

The key question in conformation sampling for template-free PSP is how to obtain a broad, sample-based representation of the vast and high-dimensional conformation spaces (and in turn the associated energy surface) and not miss possibly diverse local minima that may house near-native conformations. The response to this question traditionally has been *by the numbers*; that is, the objective becomes to generate a large number of low-energy conformations (also referred to as decoys) as a way of increasing the likelihood of having a diverse decoy dataset that covers a sufficient number of local minima possibly housing near-native conformations.

In this paper we pursue a complementary approach and propose to directly control the diversity of sampled conformations. Inspired by hard optimization problems in high-dimensional and non-linear variable spaces, we propose that conformation sampling for decoy generation is more naturally framed as a multi-objective optimization problem. We demonstrate that mechanisms inherent to evolutionary search techniques facilitate such framing and allow balancing multiple competing objectives in protein conformation sampling. We showcase an operationalization of this idea via a novel evolutionary algorithm that has high exploration capability and is additionally able to access lower-energy regions of the energy landscape of a given protein with similar or better proximity to the known native structure than state-of-the-art algorithms.

The rest of this article is organized as follows. Related work is summarized in the following section. The proposed algorithm is described in the “Methods” section and evaluated in the “Results” section. The article concludes with a summary and discussion of future directions of work in the “Conclusion” section.

### Related work

Key features are behind advances over the past decade in template-free PSP. The conformation space is simplified and reduced in dimensionality. The atoms of the side chain in each amino acid are compressed into a pseudo-atom, and the conformation variables are dihedral angles on bonds connecting modeled backbone atoms and side-chain pseudo-atoms. Note that even this representation yields hundreds of dihedral angles (thus, a conformation space of hundreds of dimensions) even for chains not exceeding 150 amino acids. Additionally, the molecular fragment replacement technique is used to discretize the conformation space by bundling backbone dihedral angles together. Values are assigned for a consecutive number of angles simultaneously according to structural pieces or fragment configurations that are pre-compiled over known native protein structures [6].

Despite these two key developments, the conformation space demands powerful optimization algorithms under the umbrella of stochastic optimization. These algorithms have to balance limited computational resources between exploration of a space through global search with exploitation of local minima in the energy surface (the conformation space lifted by the internal energy of each conformation) through local search. The common approach, in Rosetta and others [10], achieves exploitation through intensive localized MMC search, while using multi-start or random-restart for global search or exploration. There are no explicit controls in these MMC-based treatments to balance between exploration and exploitation, which is key when the search space is high-dimensional and highly non-linear (rich in local minima). Moreover, to account for the fact that computational resources may be wasted on exploiting false local minima (artifacts of the particular energy function used)^2^, the recommendation from developers is to generate a large number of decoys (e.g., run the Rosetta abinitio protocol for conformation sampling tens of thousands of times).

MMC-based treatments do not address the core issue of balancing exploration with exploitation. Evolutionary algorithms (EAs) are inherently better equipped at addressing this balance for complex optimization problems [11]. A growing body of research shows that, when injected with domain-specific insight (as in Rosetta), EAs outperform Rosetta in exploration capability [12–16]. EAs carry out stochastic optimization inspired by natural selection. In particular, in population-based EAs, a fixed-size population of individuals (conformations in our context) evolves over a number of generations. At every generation, individuals are selected to serve as parents. Selected parents are subjected to variation operators that produce new offspring. In memetic/hybrid EAs, this global search is interleaved with local search, as offspring are additionally subjected to an improvement operator, so that they can better compete with parents. A selection operator implements the concept of natural selection, as it pares down the combined parent and offspring population down to the fixed-size population. The interested reader is pointed to work in [14] for a review of EAs for template-free PSP over the years.

EAs easily allow for framing conformation sampling for template-free PSP as a multi-objective optimization problem. The latter may not seem immediately obvious, but the rise of false local minima is due to lack of knowledge on how to combine competing atomic interactions (electrostatic, hydrogen-bonding, and others) and how much to weight each category of interactions in an energy function. These categories are often conflicting; that is, a change in a conformation may cause an increase in the value of one energetic term (e.g., electrostatics) but a decrease in the value of another (e.g., hydrogen bonding). Rather than combining such terms in one energy function that is used as an aggregate optimization objective, proof-of-concept work has pursued a multi-objective optimization setting by treating different terms in an energy function as separate optimization objectives [16, 17]. It is worth noting that algorithmic ingredients in an EA (its various operators) naturally allow pursuing a multi-objective optimization treatment for decoy generation. Moreover, as we show in this paper, such mechanisms allow to control the diversity of sampled conformations and thus yield a broader, sample-based representation of the conformation space (and its energy surface).

## Methods

The proposed algorithm is a memetic EA that controls the diversity of the conformations it computes via the selection operator that determines individual survival. The algorithm builds over expertise in our laboratory on EAs for decoy generation; namely, how to inject Rosetta domain-specific insight (structure representation, molecular fragment replacement technique, and scoring functions for conformation evaluation) in evolutionary search mechanisms. The methodological contribution in this paper is a novel, sophisticated selection operator to control conformation diversity and handle conflicting optimization objectives.

### Summary of main ingredients

We provide a summary of the main computational ingredients first. The proposed EA evolves a fixed-size population of *N* conformations over generations. Great care is taken so the initial population *P*_0_ contains *N* physically-realistic, yet diverse conformations. Each conformation is initialized as an extended backbone conformation, and a series of fragment replacements randomize each conformation while adding secondary structure. This process is conducted as a Monte Carlo search, guided by two different scoring functions that first encourage avoidance of steric clashes (self-collisions) and then the formation of secondary structure.

In the proposed EA, at the beginning of each generation, all conformations in the population are selected as parents and varied so that each yields one offspring conformation. The variation makes use of the popular molecular fragment replacement technique (described in greater detail below), effectively selecting a number of consecutive dihedral angles starting at some amino acid selected at random and replacing the angles with new ones drawn from a pre-compiled fragment library. This process and the variation operator are described in greater detail below. The variation operator contributes to exploration. To additionally improve exploitation (digging deeper into the energy surface), each offspring is further subjected to an improvement operator. This operator maps each offspring to a nearby local minimum in the energy surface via a greedy local search (that again utilizes fragment replacements), detailed below. At the end of the variation and improvement operators, the algorithm has now computed *N* new (offspring) conformations that will fight for survival among one another and the *N* parent conformations. The winners constitute the next population.

We now describe each of the operators in further detail.

### Fragment replacement

In molecular fragment repacement, an amino acid in the segment [1,*l*−*f*+1] (where *l* is the number of amino acids in the protein chain) over the chain of amino acids is selected at random, effectively picking at random a fragment [*i*,*i*+*f*−1] of *f* consecutive amino acids in the sequence. This sequence of amino acids exists in some fragment configuration in some current conformation *C*_curr_. The entire configuration of 3×*f* backbone dihedral angles (*ϕ*,*ψ*, and *ω* per amino acid) in *C*_curr_ is replaced with a new configuration of 3×*f* backbone dihedral angles to obtain *C*_new_. The new configuration is obtained from pre-compiled fragment libraries. These libraries are computed over known native structures of proteins (deposited, for instance, in the Protein Data Bank) and are organized in such a way that a query with the amino-acid sequence of a fragment returns 200 configurations; one is selected at random to replace the configuration in the selected fragment in *C*_curr_. The described process is the molecular fragment replacement in Rosetta. The reader is referred to Ref. [6] for further information on fragment libraries.

### Initial population operator

Recall that a population contains a fixed number of conformations *N*. Given the amino-acid sequence of *l* amino acids, the Pose construct of the Rosetta framework is utilized to obtain an extended chain of backbone atoms, with the side-chain of each amino acid reduced to a centroid pseudo-atom (this is known as the centroid representation in Rosetta). This process is repeated *N* times to obtain *N* (identical) extended conformations. Each extended conformation is then subjected to two consecutive stages of local search. Each one is implemented as an MMC search, but the stages use different scoring functions and different values for the scaling parameter *α* that controls the acceptance probability in the Metropolis criterion. In both stages, an MC move is a fragment replacement; a fragment of length 9 (9 consecutive amino acids) is selected at random over the chain of amino acids and replaced with a fragment configuration drawn at random from 9 amino-acid (aa) long fragment libraries. The latter are pre-built given a target sequence by making use of the online Robetta fragment server [6].

In the first stage, the goal is to randomize each extended chain via fragment replacements but still avoid self collisions. The latter are penalized in the score0 scoring function, which is a Rosetta scoring function that consists of only a soft steric repulsion. This scoring function is utilized in stage one to obtain a diverse population of random conformations free of self collisions. A scaling parameter *α*=0 is used in the Metropolis criterion; this effectively sets the acceptance probability to 0, which guarantees that a move is only accepted if it lowers score0. This strict constraint is necessary to avoid carrying through self-colliding conformations.

In the second stage, the goal changes from obtaining randomized, collision-free conformations to conformations that resemble protein structures in that they have secondary structure elements that are packed rather than stretched out in space. This is achieved by switching from score0 to score1, which imposes more constraints than collision avoidance and allows formation of secondary structure. In addition, the scaling parameter is set to a higher value of 2, which increases the acceptance probability, increasing the diversity of conformations. This stage, also implemented as an MMC search where moves are fragment replacements, proceeds on a conformation until *l* consecutive moves (*l* is number of amino acids in a given protein sequence) fail per the Metropolis criterion. We note that score0 and score1 are members of a suite of Rosetta scoring functions that are weighted sums of 13 distinct energy terms. The process employed in the initial population (utilizing fragment length of 9 and different scoring functions at different substages) mirrors that in Rosetta (though the length of the MMC trajectories in the substages in the simulated annealing algorithm employed for decoy generation in Rosetta is much longer). The final ensemble of conformations obtained by the initial population operator now contains credible, protein-like conformations.

### Variation operator

The variation operator is applied onto a parent individual to obtain offspring. This operator implements asexual reproduction/mutation, making use of fragment replacement to vary a parent and obtain a new, offspring conformation. We note that in the variation operator, one does not want to institute too much of a (structural) change from the parent in the offspring, so that good properties of the parent are transferred to the offspring, but enough change to obtain a conformation different from the parent. For this reason, a fragment length *f*=3 is used in the variation operator. Note that the fragment replacement in the variation operator is not in the context of some MMC search; that is, one fragment replacement is carried out, and the result is accepted, yielding an offspring conformation obtained from a thus-varied parent.

### Improvement operator

This operator maps an offspring to a nearby local minimum via a greedy local search that resembles stage two in the initial population operator. The search carries out fragment replacements (utilizing *f*=3) that terminates on an offspring when *k* consecutive moves fail to lower energy. The latter is measured via Rosetta’s score3. This scoring function upweights energetic constraints (terms) that favor formation of compact tertiary structures [18]. The utilization of score3 in the proposed algorithm mirrors the fact that in Rosetta, the majority of the search is done with score3. That is, most of the computational budget (in terms of fitness evaluations) is expended on the local improvement operator.

### Selection operator

The selection operator is the mechanism leveraged to pursue a multi-objective optimization setting and directly control the diversity of computed conformations. We first describe how the selection operator allows a multi-objective optimization setting.

#### Multi-objective optimization under Pareto dominance

Let us consider that a certain number of optimization objectives is provided along which to compare conformations. A conformation *C*_*a*_ is said to *dominate* another conformation *C*_*b*_ if the value of each optimization objective in *C*_*a*_ is lower than the value of that same objective in *C*_*b*_; this is known as strong dominance. If equality is allowed, the result is soft dominance. The proposed algorithm makes use of strong dominance. Utilizing the concept of dominance, one can measure the number of conformations that dominate a given conformation *C*_*b*_. This measure is known as *Pareto rank* (PR) or, equivalently, *domination count*. In contrast, the number of conformations dominated by a given conformation *C*_*a*_ is known as the *Pareto count* (PC) of *C*_*a*_. If no conformation in a set dominates a given conformation *C*_*b*_, then *C*_*b*_ has a domination count (PR) of 0 and is said to be *non-dominated*. Non-dominated conformations constitute the *Pareto front*.

The concept of Pareto dominance can be operationalized in various ways. In early proof-of-concept work [16, 17], the Rosetta score4 (which includes both short-range and long-range hydrogen bonding terms) was divided into three optimization objectives along which parents and offspring can be compared in the selection operator: short-range hydrogen bonds (objective 1), long-range hydrogen bonds (objective 2), and everything else (summed together in objective 3). This categorization recognizes the importance of hydrogen bonds for formation of native structure [18]. Using these three objectives, work in [16] utilizes only PR in the selection operator, first sorting the *N* parent and *N* offspring conformations from low to high PR, and then further sorting conformations with the same PR from low to high score4 (total energy that sums all three objectives). PC can be additionally considered to obtain a sorted order, as in [17]. Conformations with the same PR are sorted from high to low PC, and conformations with the same PC are further sorted from low to high score4. The selection operator then selects the top *N* conformations (out of the combined 2*N* conformations of parents and offspring) according to the resulting sorted order.

##### Non-dominated Fronts

The proposed algorithm truly considers a multi-objective setting and does not utilize an aggregate energy value (the sum of the objectives). Specifically, the algorithm considers non-dominated fronts in its selection operator. A fast, non-dominated sorting algorithm (originally proposed in [19]) is used to generate these fronts as follows. All the conformations in the combined parent and offspring population that have a domination count of 0 (thus, are non-dominated) make up the first non-dominated front *F*_1_. Each subsequent, non-dominated front *F*_*i*_ is generated as follows. For each conformation *C*∈*F*_*i*−1_, the conformations dominated by *C* constitute the set *S*_*C*_. The domination count of each member in *S*_*C*_ is decremented by 1. Conformations in *S*_*C*_ that have their domination count reduced to 0 make up the subsequent, non-dominated front *F*_*i*_. This process of generating non-dominated fronts terminates when the total number of conformations over the generated fronts equals or exceeds the population size *N*. In this way, the selection operator is accumulating enough good-quality conformations from which it can further draw based on additional non-energy based objectives. Moreover, this allows generating Pareto-optimal solutions over the generations and achieving better convergence to the true, Pareto-optimal set.

#### Density-based conformation diversity

Borrowing from evolutionary computation research [19] on optimization problems of few variables ranging from 1 to 30 (as opposed to hundreds of variables in our setting), we leverage crowding distance to retain diverse conformations. Crowding distance estimates the density of the conformations in the population space and guides the selection process over generations towards less crowded regions [19]. We use the crowding distance assignment technique to compute the average distance of a conformation from other conformations in the same non-dominated front along each of the optimization objectives. First, the crowding distance of each conformation is initialized to 0. Then, for each objective, conformations are sorted based on their corresponding score (value of that objective) in ascending order and assigned infinite distance value to conformations with the highest and lowest scores; this ensures that conformations with the highest and lowest scores (effectively constituting the boundaries of the population space) are always selected. For all other conformations *C*, the absolute normalized difference in scores between the two closest conformations on either side of *C* is added to the crowding distance. Finally, when all the objectives are considered, the crowding distance of a conformation is the sum of the individual distances along each objective.

#### Putting it all together: Conformation diversity in a multi-objective optimization setting

To obtain the next population, the selection operator selects *r* conformations from the non-dominated fronts *F*_1_,*F*_2_,…,*F*_*t*_ sequentially, where *r* is $\sum _{i \in \{1, 2, \ldots, t\}}F_{i}$ until *r*+|*F*_*t*+1_| reaches or exceeds *N*. If *r*<*N*, which is usually the case, the crowding distance of conformations in *F*_*t*+1_ is computed and used to sort them in descending order. The selection operator then selects the top *N*−*r* conformations in this order.

It is worth noting that in our earlier operationalizations of multi-objective optimization for template-free PSP, all conformations ever computed were retained for the calculation of PR and PC values for each conformation. This introduces a significant computational overhead, which the proposed algorithm circumvents. The proposed algorithm instead uses only the current combined population of parents and offspring to perform selection, thus saving such overhead.

### Implementation details

The population size is *N*=100 conformations, in keeping with earlier work on multi-objective EAs. Instead of imposing a bound on the number of generations, the proposed algorithm is executed for a fixed budget of 10,000,000 energy evaluations. The algorithm is implemented in Python and interfaces with the PyRosetta library. The algorithm takes 1−4 h on one Intel Xeon E5-2670 CPU with 2.6GHz base processing speed and 64GB of RAM. The range in running time depends primarily on the length of the protein. As further described in the “Results” section, the algorithm is run 5 times on a test case (a target amino-acid sequence) to remove differences due to stochasticity.

## Results

### Experimental setup

The evaluation is carried out on two datasets, a benchmark dataset of 20 proteins of varying folds (*α*,*β*,*α*+*β*, and *coil*) and lengths (varying from 53 to 146 amino acids), and a dataset of 10 hard, free-modeling targets from the Critical Assessment of protein Structure Prediction (CASP) community experiment. The first dataset was first presented partially in [20] and then enriched with more targets in [12, 13, 16, 21, 22]. Our second dataset consists of 10 free-modeling domains from CASP12 and CASP13.

The proposed algorithm is compared with Rosetta’s decoy sampling algorithm, a memetic EA that does not utilize multi-objective optimization [15], and two other memetic EAs that do so (one utilizing only Pareto Rank [16], and the other utilizing both Pareto Rank and Pareto Count [17], as described in the previous section). We will correspondingly refer to these algorithms as Rosetta, mEA, mEA-PR, and mEA-PR+PC. To aid in the comparisons, we will refer to the algorithm proposed in this paper as Evo-Diverse. This comparison allows us to isolate the impact of the selection operator in Evo-Diverse over those in mEA-PR, and mEA-PR+PC, as well as point to the impact of the multi-objective setting (in comparison with mEA) and the evolutionary computation framework overall (in comparison with Rosetta). Each of these algorithms is run 5 times on each target sequence, and what is reported is their best performance over all 5 runs combined. Each run continues for a fixed computational budget of 10*M* energy evaluations.

In keeping with published work on EAs [14], performance is measured by the lowest energy ever reached and the lowest distance ever reached to the known native structure of a target under consideration. The former measures the exploration capability. Since lower energies do not necessarily correlate with proximity to the native structure, it is important to also measure the distance of each decoy to a known native structure. We do so via a popular dissimilarity metric, least root-mean-squared-deviation (lRMSD) [23]. lRMSD first removes differences due to rigid-body motions (whole-body translation and rotation in three dimensions), and then averages the summed Euclidean distance of corresponding atoms in two conformations over the number of atoms compared. Typically, in template-free PSP, the comparison focuses on the main carbon atom of each amino acid (the CA atoms). It is worth noting that lRMSD is non-descriptive above 8Å and increases with sequence/chain length. An RMSD within 5−6Å is considered to have captured the native structure. In addition to lRMSD, our evaluation on the CASP12 and CASP13 dataset includes two additional measures, the “Template Modeling Score” (TM-score) [24] and the “Global Distance Test - Total Score” (GDT_TS) [25, 26]. Both metrics produce a score between 0 and 1, where a score of 1 suggests a perfect match. A higher score indicates a better proximity. In practice, TM-scores and GDT_TS scores of 0.5 and higher are indicative of good predictions/models.

To carry out a principled comparison, we evaluate the statistical significance of the presented results. We use Fisher’s [27] and Barnard’s [28] exact tests over 2x2 contingency matrices keeping track of the particular performance metric under comparison. Fisher’s exact test is conditional and widely adopted for statistical significance. Barnard’s test is unconditional and generally considered more powerful than Fisher’s test on 2x2 contingency matrices. We use 2-sided tests to determine which algorithms do not have similar performance and 1-sided tests to determine if Evo-Diverse performs significantly better than the other algorithms under comparison.

### Comparative analysis on benchmark dataset

**Fig. 1:**
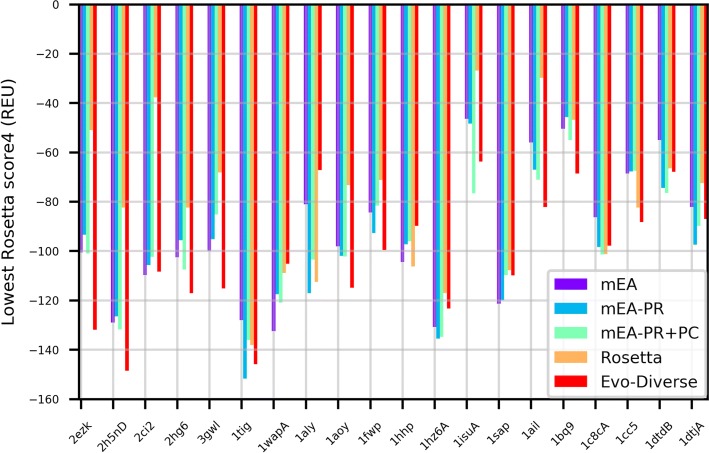
The lowest Rosetta score4 (measured in Rosetta Energy Units – REUs) to a given native structure obtained over 5 runs of each algorithm on each of the 20 test cases of the benchmark dataset is shown here, using different colors to distinguish the algorithms under comparison

**Fig. 2:**
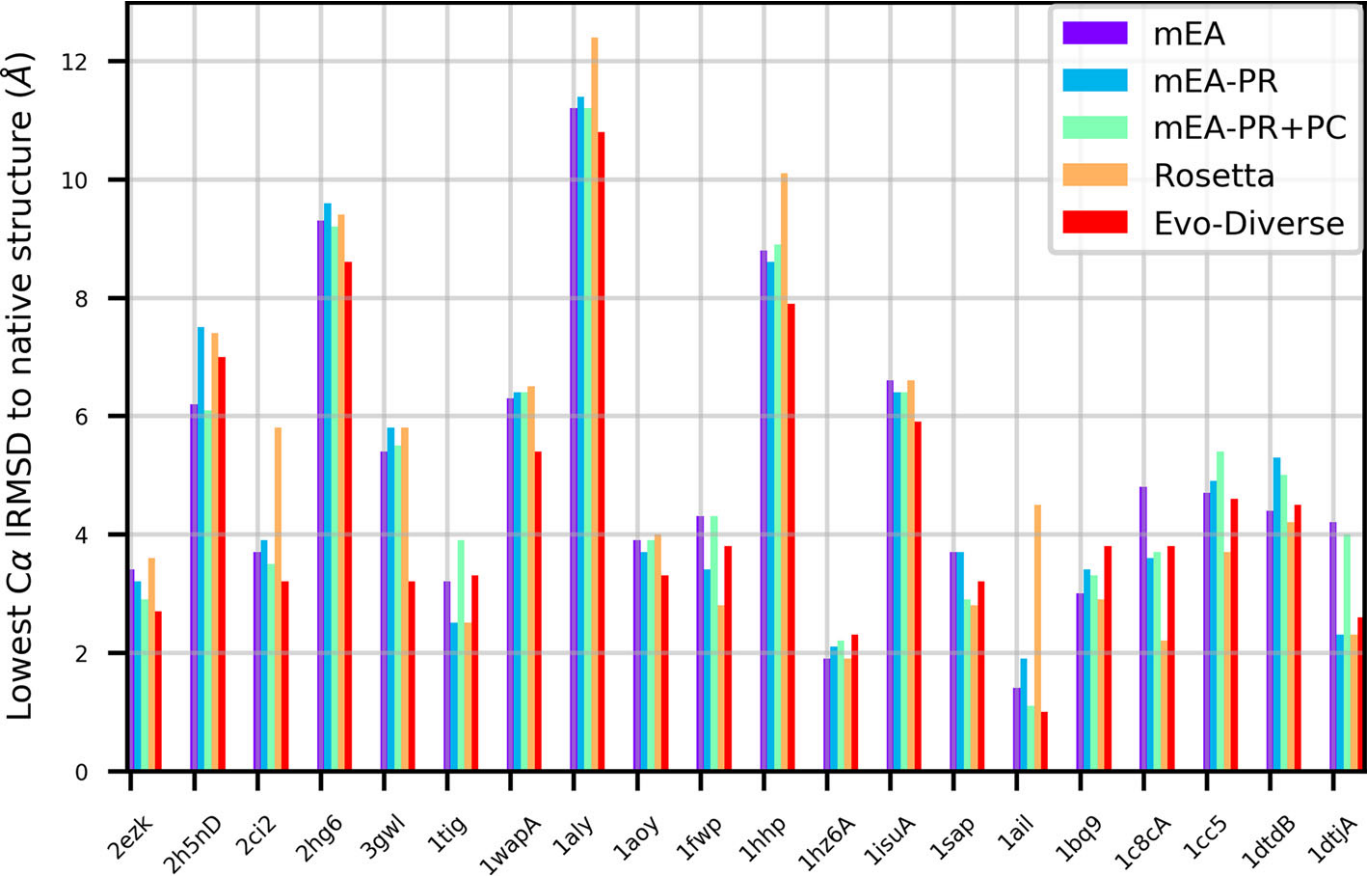
The lowest lRMSD (measured in Angstroms – Å) to a given native structure obtained over 5 runs of each algorithm on each of the 20 test cases of the benchmark dataset is shown here, using different colors to distinguish the algorithms under comparison

Figure 1 shows the lowest energy obtained over combined 5 runs of mEA, mEA-PR, mEA-PR+PC, Rosetta, and Evo-Diverse for each of the 20 target proteins; the latter are denoted on the x axis by the Protein Data Bank (PDB) [2] identifier (ID) of a known native structure for each target. Figure 2 presents the comparison in terms of the lowest lRMSD achieved on each of the test cases. Color-coding is used to distinguish the algorithms from one another.

A summary of comparative observations is presented in Table 1. Table 1(a) shows that lowest energy is achieved by Evo-Diverse in 9/20 of the test cases over the other algorithms; in comparison, mEA-PR achieves the lowest energy in 4/20, mEA and mEA-PR+PC in 3/20, and Rosetta in only 1 case. In a head-to-head comparison, Evo-Diverse bests each of the other algorithms in a comparison of lowest energy. Table 1(b) shows that lowest lRMSD is achieved by Evo-Diverse in 10/20 test cases over the other algorithms; in comparison, mEA-PR achieves the lowest energy in 2/20, mEA and mEA-PR+PC in 1/20, and Rosetta in 9 cases. In a head-to-head comparison, Evo-Diverse bests each of the other algorithms in a comparison of lowest lRMSD, as well.

**Table 1:** Comparison of the number of test cases of the benchmark dataset on which the algorithms achieve the lowest energy value. Comparison of the number of test cases of the benchmark dataset on which the algorithms achieve the lowest lRMSD value

(a)
**Evo-Diverse vs. others**: 9 vs. 3 (mEA), 4 (mEA-PR), 3 (mEA-PR+PC), and 1 (Rosetta)
**Evo-Diverse vs. mEA**: 14 vs. 6
**Evo-Diverse vs. mEA-PR**: 11 vs. 9
**Evo-Diverse vs. mEA-PR+PC**: 12 vs. 8
**Evo-Diverse vs Rosetta**: 16 vs. 4
(b)
**Evo-Diverse vs. others**: 10 vs. 1 (mEA), 2 (mEA-PR), 1 (mEA-PR+PC), and 9 (Rosetta)
**Evo-Diverse vs. mEA**: 15 vs. 5
**Evo-Diverse vs. mEA-PR**: 14 vs. 6
**Evo-Diverse vs. mEA-PR+PC**: 15 vs. 5
**Evo-Diverse vs Rosetta**: 11 vs. 9

The above comparisons are further strengthened via statistical analysis. Table 2(a) shows the *p*-values obtained in 1-sided statistical significance tests that pitch Evo-Diverse against each of the other algorithms (in turn), evaluating the null hypothesis that Evo-Diverse performs similarly or worse than its counterpart under comparison, considering two metrics, achieving the lowest energy in each test case, and achieving a lower (lowest) energy on each test case that its current counterpart. Both Fisher’s and Barnard’s test are conducted, and *p*-values less than 0.05 (which reject the null hypothesis) are marked in bold. Table 2(a) shows that the null hypothesis is rejected in most of the comparisons; Evo-Diverse performs better than mEA and Rosetta; the performance over mEA-PR and mEA-PR+PC is not statistically significant.

**Table 2:** Comparison of Evo-Diverse to other algorithms on lowest energy via 1-sided Fisher’s and Barnard’s tests on the benchmark dataset. Top panel evaluates the null hypothesis that Evo-Diverse does not achieve the lowest energy, considering each of the other four algorithms in turn. The bottom panel evaluates the null hypothesis that Evo-Diverse does not achieve a lower lowest energy value in comparison to a particular algorithm, considering each of the four other algorithms in turn. Comparison of Evo-Diverse to other algorithms on lowest lRMSD via 1-sided Fisher’s and Barnard’s tests on the benchmark dataset. Top panel evaluates the null hypothesis that Evo-Diverse does not achieve the lowest lRMSD, considering each of the other four algorithms in turn. The bottom panel evaluates the null hypothesis that Evo-Diverse does not achieve a lower lowest lRMSD value in comparison to a particular algorithm, considering each of the four other algorithms in turn

Test	mEA	mEA-PR	mEA-PR+PC	Rosetta
(a)				
Best lowest energy				
Fisher’s	**0.04118**	0.088	**0.04118**	**0.004181**
Barnard’s	**0.02489**	0.05368	**0.02489**	**0.001879**
Better lowest energy				
Fisher’s	**0.01282**	0.3762	0.1715	**0.00018**
Barnard’s	**0.008299**	0.3179	0.1341	**0.00009139**
(b)				
Best lowest lRMSD				
Fisher’s	**0.001671**	**0.006907**	**0.001671**	0.5
Barnard’s	**0.000702**	**0.003284**	**0.000702**	0.4373
Better lowest lRMSD				
Fisher’s	**0.001924**	**0.01282**	**0.001924**	0.3762
Barnard’s	**0.001118**	**0.008299**	**0.001118**	0.3179

*p*-values less than 0.05 are marked in bold

Table 2(b) shows the *p*-values obtained in 1-sided statistical significance tests that pitch the performance of Evo-Diverse against each of the other algorithms (in turn), evaluating the null hypothesis that Evo-Diverse performs similarly or worse than its counterpart under comparison, considering two metrics, achieving the lowest lRMSD in each test case, and achieving a lower (lowest) lRMSD on each test case than its current counterpart. Both Fisher’s and Barnard’s test are conducted, and *p*-values less than 0.05 (rejecting the null hypothesis) are in bold. Table 2(b) shows that the null hypothesis is rejected in most tests; Evo-Diverse outperforms all algorithms except for Rosetta.

Table 3(a) shows the *p*-values obtained in 2-sided statistical significance tests that pitch Evo-Diverse against each of the other algorithms (in turn), evaluating the null hypothesis that Evo-Diverse performs similarly to its counterpart under comparison, considering two metrics, achieving the lowest energy in each test case, and achieving a lower (lowest) energy on each test case than its current counterpart. Both Fisher’s and Barnard’s test are conducted, and *p*-values less than 0.05 (which reject the null hypothesis) are marked in bold. Table 2(a) shows that the null hypothesis is rejected in most of the comparisons; Evo-Diverse does not perform similarly to mEA and Rosetta; the dissimilarity of performance compared to mEA-PR and mEA-PR+PC is not statistically significant at 95% confidence level. Similarly, Table 3(b) shows the *p*-values obtained in 2-sided statistical significance tests that now consider the lowest lRMSD instead of lowest energy. Table 3(b) shows that the null hypothesis is rejected in most tests; Evo-Diverse does not perform similarly to all algorithms except for Rosetta at 95% confidence level.

**Table 3:** Comparison of Evo-Diverse to other algorithms on lowest energy via 2-sided Fisher’s and Barnard’s tests on the benchmark dataset. Top panel evaluates the null hypothesis that Evo-Diverse achieves similar performance on reaching the lowest energy, considering each of the other four algorithms in turn. The bottom panel evaluates the null hypothesis that Evo-Diverse achieves similar performance on reaching a lower lowest energy value in comparison to a particular algorithm, considering each of the four other algorithms in turn. Comparison of Evo-Diverse to other algorithms on lowest lRMSD via 2-sided Fisher’s and Barnard’s tests on the benchmark dataset. Top panel evaluates the null hypothesis that Evo-Diverse achieves similar performance on reaching the lowest lRMSD, considering each of the other four algorithms in turn. The bottom panel evaluates the null hypothesis that Evo-Diverse achieves similar performance on reaching a lower lowest lRMSD value in comparison to a particular algorithm, considering each of the four other algorithms in turn

Test	mEA	mEA-PR	mEA-PR+PC	Rosetta
(a)				
Best lowest energy				
Fisher’s	0.08236	0.176	0.08236	**0.008362**
Barnard’s	**0.04977**	0.1074	**0.04977**	**0.003759**
Better lowest energy				
Fisher’s	**0.02564**	0.7524	0.3431	**0.00036**
Barnard’s	**0.0166**	0.6358	0.2682	**0.0001828**
(b)				
Best lowest lRMSD				
Fisher’s	**0.003342**	**0.01381**	**0.003342**	1
Barnard’s	**0.001404**	**0.006567**	**0.001404**	0.8746
Better lowest lRMSD				
Fisher’s	**0.003848**	**0.02564**	**0.003848**	0.7524
Barnard’s	**0.002236**	**0.0166**	**0.002236**	0.6358

*p*-values less than 0.05 are marked in bold

Taken altogether, these results indicate that Evo-Diverse has a high exploration capability, decidedly outperforming mEA and Rosetta in terms of its ability to wisely use a fixed computational budget to reach lower-energy levels, and performing similarly or better than mEA-PR and mEA-PR+PC. The latter result is not surprising, as mEA-PR, mEA-PR+PC, and Evo-Diverse use a multi-objective optimization framework, which delays a premature convergence, thus allowing them to reach lower energies within the same computational budget provided to mEA and Rosetta. Interestingly though, the head-to-head lRMSD comparisons show that, while mEA-PR and mEA-PR+PC achieve lower energies than Rosetta, this does not help them achieve the same performance as Rosetta in terms of lowest lRMSDs. In contrast, Evo-Diverse effectively retains the best of both. It is able to reach lower energies than Rosetta and comparable or lower lRMSDs than Rosetta, thus constituting a clear advantage over the current state-of-the-art multi-objective optimization EAs.

**Fig. 3:**
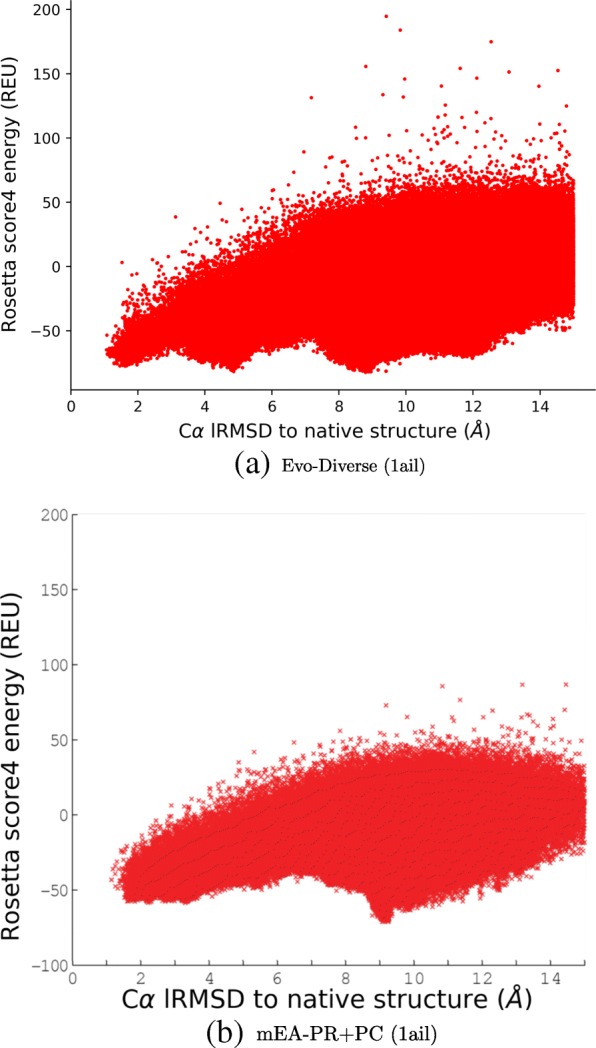
Decoys are shown by plotting their Rosetta score4 vs. their CA lRMSD from the native structure (PDB ID in parentheses) to compare the landscape probed by different algorithms (Evo-Diverse (**a**), mEA-PR+PC (**b**)) for the target with known native structure under PDB id 1ail

**Fig. 4:**
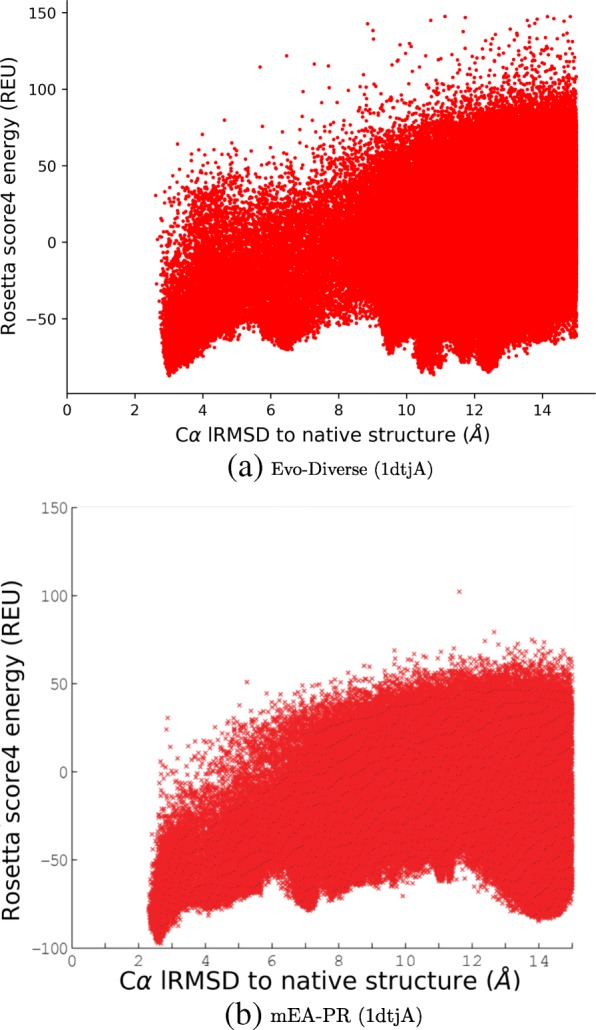
Decoys are shown by plotting their Rosetta score4 vs. their CA lRMSD from the native structure (PDB ID in parentheses) to compare the landscape probed by different algorithms (Evo-Diverse (**a**), mEA-PR (**b**)) for the target with known native structure under PDB id 1dtjA

When analyzing the performance of decoy generation algorithms, it is additionally informative to visualize the energy landscape that they probe one decoy at a time. We do so by plotting decoy-energy pairs, representing a decoy with its lowest lRMSD coordinate to the known native structure of each test case. Figures 3 and 4 juxtapose such landscapes for two selected test cases, the protein with known native structure under PDB ID 1ail, and that with known native structure under PDB ID 1dtjA, respectively.

The comparison is limited here to landscapes probed by Evo-Diverse, mEA-PR, and mEA-PR+PC, as prior work comparing mEA-PR and mEA-PR+PC to Rosetta and mEA shows that these two algorithms achieve better funneling (better correlation between low energies and low lRMSDs to the native structure), and that mEA-PR+PC does so the best for 1ail, while mEA-PR does so for 1dtjA.

Figure 3 shows that Evo-Diverse reveals better funneling of the landscape than mEA-PR+PC (higher correlation between low energies and low lRMSDs) and multiple non-native local minima, visually confirming its high exploration capability. Figure 4 shows that Evo-Diverse and mEA-PR reveal similar correlation between low energies and low lRMSDs (higher than both Rosetta and mEA) and multiple non-native local minima.

**Fig. 5:**
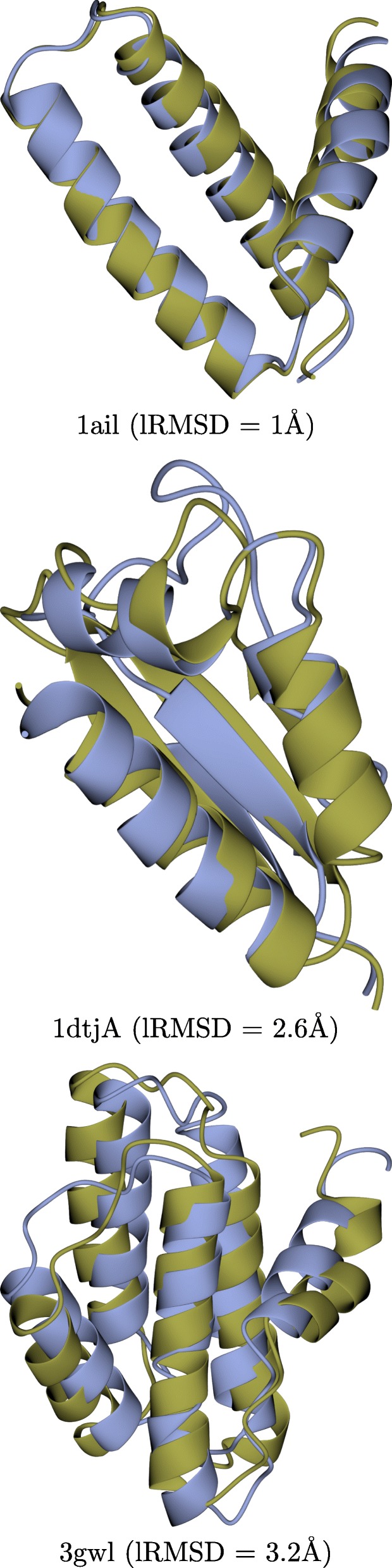
The decoy obtained by Evo-Diverse that is closest to the native structure is shown for three selected cases, the protein with known native structure under PDB ID 1ail (top), 1dtjA (middle), and 3gwl (bottom). The Evo-Diverse decoy is in blue, and the known native structure is in orange

Figure 5 superimposes the best decoy (lowest lRMSD to the known native structure) over the known native structure for three selected proteins (PDB IDs 1ail, 1dtjA, and 3gwl). Rendering is performed with the CCP4mg molecular graphics software [29]. In the case of 1ail, Evo-Diverse obtains the lowest lRMSD to the native structure (1Å). On 1dtjA, Evo-Diverse reaches a similar lowest lRMSD (2.6Å) as Rosetta and mEA-PR (confirmed in Fig. 2). On 3gwl, Evo-Diverse achieves a dramatic improvement of lowest lRMSD to the native structure over all other algorithms; while none of the other algorithms reach below 5Å, Evo-Diverse reaches 3.2Å, almost a 2Å improvement.

### Comparative analysis on CASP 12-13 dataset

Table 4 shows the lowest energy and the average energy of the 10 best decoys obtained by Evo-Diverse and Rosetta on each of the 10 target domains denoted by their identifiers in column 1. The lower energy values between the two algorithms on each target domain are marked in bold. Table 4 shows that lower energy values are obtained by Evo-Diverse in 7/10 cases compared to Rosetta’s 3/10 cases. When the average of the best 10 decoys is considered instead, Evo-Diverse achieves lower energy values in 8/10 cases compared to Rosetta’s 2/10 cases.

**Table 4:** Comparison of energy of the lowest energy decoy and average energy of the 10 best decoys (measured in Rosetta Energy Units – REUs) obtained by each algorithm on each of the 10 CASP domains

			Lowest energy		Avg. of the best 10	
Domain	CASP	Length	Rosetta	Evo-Diverse	Rosetta	Evo-Diverse
T1008-D1	13	77	− 164.2	**−** **1** **6** **6** **.** **4**	− 162	**−** **1** **6** **6** **.** **3**
T0957s1-D1	13	108	**−** **1** **2** **1** **.** **5**	− 112.6	**−** **1** **1** **5**	− 112.6
T0892-D2	12	110	− 101.8	**−** **1** **1** **2** **.** **3**	− 94.1	**−** **1** **1** **2** **.** **3**
T0953s2-D3	13	93	− 53.1	**−** **6** **7** **.** **6**	− 49.8	**−** **6** **6** **.** **3**
T0960-D2	13	84	− 79.7	**−** **8** **2** **.** **3**	− 79.4	**−** **8** **2** **.** **3**
T0898-D2	12	55	− 65.5	**−** **6** **6** **.** **7**	− 62.8	**−** **6** **6** **.** **7**
T0859-D1	12	129	**−** **9** **9** **.** **5**	− 85.6	**−** **9** **0** **.** **7**	− 85.6
T0897-D1	12	138	− 141.4	**−** **1** **4** **7** **.** **4**	− 137.4	**−** **1** **4** **7** **.** **4**
T0886-D1	12	69	**−** **8** **9** **.** **2**	− 85.4	− 84	**−** **8** **5** **.** **4**
T0953s1-D1	13	67	− 51.8	**−** **5** **9**	− 49.1	**−** **5** **9**

Lowest values for each target are marked in bold

The above comparisons are further strengthened via statistical analysis. Table 8(a) shows the *p*-values obtained in 1-sided statistical significance tests that pitch Evo-Diverse against Rosetta, evaluating the null hypothesis that Evo-Diverse performs similarly or worse than Rosetta. Both Fisher’s and Barnard’s test are conducted, and *p*-values less than 0.05 (which reject the null hypothesis) are marked in bold. Table 8(a) shows that the null hypothesis is rejected when the average of the best 10 decoys is considered, and Evo-Diverse performs significantly better than Rosetta with 95% confidence. When the focus is on the lowest energy reached, the performance improvement of Evo-Diverse over Rosetta is not statistically significant at 95% confidence level, although *p*-values are very close to the 0.05 threshold.

**Fig. 6:**
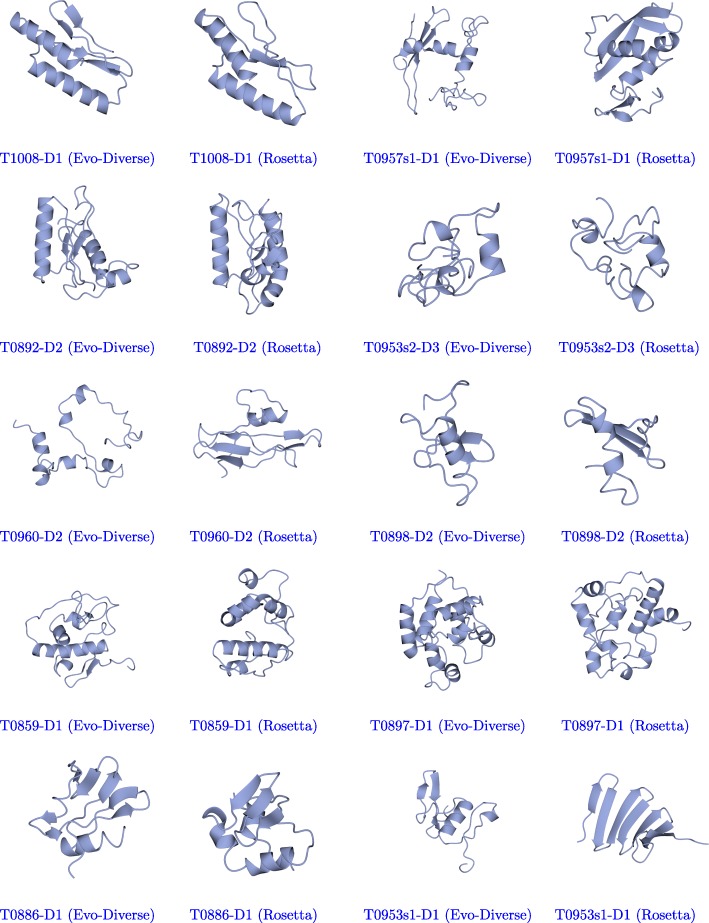
The best (lowest lRMSD to the known native structure) decoy obtained by Evo-Diverse (left) and Rosetta (right) is shown for each of the domains of the CASP dataset

Table 5 shows the lowest lRMSD to the native structure and the average lRMSD of the 10 best decoys obtained by Evo-Diverse and Rosetta on each of the 10 target domains denoted by their identifiers in column 1. The lower lRMSD values between the two algorithms on each target domain are marked in bold. Table 4 shows that lower lRMSDs are obtained by Evo-Diverse in 6/10 cases compared to Rosetta’s 4/10 cases. When the average of the best-lRMSD 10 decoys is considered, Evo-Diverse achieves lower lRMSD in 9/10 cases compared to 2/10 cases of Rosetta. Figure 6 shows the best decoy (lowest lRMSD to the known native structure) obtained on each target domain by Evo-Diverse and Rosetta. Rendering is performed with the CCP4mg molecular graphics software [29].

**Table 5:** Comparison of lRMSD to the native structure of the lowest lRMSD decoy and average lRMSD to the native of the 10 best decoys (measured in Angstroms – Å) obtained by each algorithm on each of the 10 CASP domains

			Lowest lRMSD		Avg. of the best 10	
Domain	CASP	Length	Rosetta	Evo-Diverse	Rosetta	Evo-Diverse
T1008-D1	13	77	**3** **.** **2**	3.5	**3** **.** **4**	3.8
T0957s1-D1	13	108	**6** **.** **9**	7.1	8.1	**7** **.** **6**
T0892-D2	12	110	8	**7** **.** **4**	8.5	**7** **.** **6**
T0953s2-D3	13	93	8.7	**7** **.** **9**	9.3	**8** **.** **3**
T0960-D2	13	84	**7** **.** **2**	7.3	**7** **.** **6**	**7** **.** **6**
T0898-D2	12	55	6.5	**5** **.** **9**	6.7	**6** **.** **3**
T0859-D1	12	129	10.6	**9** **.** **4**	11.3	**9** **.** **9**
T0897-D1	12	138	**9**	9.3	10.8	**9** **.** **9**
T0886-D1	12	69	6.3	**6** **.** **2**	6.8	**6** **.** **6**
T0953s1-D1	13	67	7	**5** **.** **7**	7.4	**6** **.** **1**

Lowest values for each target are marked in bold

The above comparisons are further strengthened via statistical analysis. Table 8(b) shows the *p*-values obtained in 1-sided statistical significance tests that pitch Evo-Diverse against Rosetta, evaluating the null hypothesis that Evo-Diverse performs similarly or worse than Rosetta. Again, both Fisher’s and Barnard’s test are conducted, and *p*-values less than 0.05 (which reject the null hypothesis) are marked in bold. Table 8(b) shows that the null hypothesis is rejected when the average of the best 10 decoys is considered and Evo-Diverse performs significantly better than Rosetta with 95% confidence. When the focus is on the lowest lRMSD reached, the performance improvement of Evo-Diverse over Rosetta is not statistically significant at 95% confidence level.

Table 6 shows the highest TM-score to the native structure and the average TM-score of the 10 best (in terms of TM-scores) decoys obtained by Evo-Diverse and Rosetta on each of the 10 target domains denoted by their identifiers in column 1. The higher TM-score values between the two algorithms on each target domain are marked in bold. Table 6 shows that higher TM-scores are obtained by Evo-Diverse and Rosetta on 5/10 cases. When the focus is on the average TM-score of the best (in terms of TM-scores) 10 decoys is considered, Evo-Diverse achieves higher TM-score in 6/10 cases compared to Rosetta’s 5/10.

**Table 6:** Comparison of TM-score of the highest TM-score decoy and average TM-score of the 10 best decoys obtained by each algorithm on each of the 10 CASP domains

			Highest TM-score		Avg. of the best 10	
Domain	CASP	Length	Rosetta	Evo-Diverse	Rosetta	Evo-Diverse
T1008-D1	13	77	**0** **.** **6** **1**	0.59	**0** **.** **5** **7**	0.55
T0957s1-D1	13	108	**0** **.** **4** **9**	0.42	**0** **.** **4** **2**	0.40
T0892-D2	12	110	0.45	**0** **.** **5** **0**	0.42	**0** **.** **4** **7**
T0953s2-D3	13	93	**0** **.** **2** **8**	0.25	**0** **.** **2** **5**	**0** **.** **2** **5**
T0960-D2	13	84	0.37	**0** **.** **3** **9**	0.35	**0** **.** **3** **8**
T0898-D2	12	55	**0** **.** **3** **9**	0.37	**0** **.** **3** **7**	0.36
T0859-D1	12	129	0.30	**0** **.** **3** **4**	0.29	**0** **.** **3** **3**
T0897-D1	12	138	0.35	**0** **.** **3** **6**	0.31	**0** **.** **3** **2**
T0886-D1	12	69	0.42	**0** **.** **4** **5**	0.40	**0** **.** **4** **1**
T0953s1-D1	13	67	**0** **.** **4** **7**	0.41	**0** **.** **4** **3**	0.39

Highest values for each target are marked in bold

Table 8(c) shows the *p*-values obtained in 1-sided statistical significance tests that pitch Evo-Diverse against Rosetta, evaluating the null hypothesis that Evo-Diverse performs similarly or worse than Rosetta. Both Fisher’s and Barnard’s test are conducted, and *p*-values less than 0.05 (which reject the null hypothesis) are marked in bold. Table 8(c) shows that the null hypothesis is not rejected with 95% confidence and the performance improvement of Evo-Diverse over Rosetta is not statistically significant.

Table 7 shows the highest GDT_TS score to the native structure and the average GDT_TS score of the 10 best (in terms of GDT_TS scores) decoys obtained by Evo-Diverse and Rosetta on each of the 10 target domains denoted by their identifiers in column 1. The higher GDT_TS scores between the two algorithms on each target domain are marked in bold. Table 7 shows that higher values (on both the highest GDT_TS score and the average GDT_TS score over the 10 best decoys) are achieved by Evo-Diverse in 6/10 cases compared to Rosetta’s 5/10.

**Table 7:** Comparison of GDT_TS score of the highest GDT_TS score decoy and average GDT_TS score of the 10 best decoys obtained by each algorithm on each of the 10 CASP domains

			Highest GDT_TS score		Avg. of the best 10	
Domain	CASP	Length	Rosetta	Evo-Diverse	Rosetta	Evo-Diverse
T1008-D1	13	77	**0** **.** **6** **2**	0.61	**0** **.** **6** **1**	0.58
T0957s1-D1	13	108	**0** **.** **4** **3**	0.39	**0** **.** **3** **9**	0.37
T0892-D2	12	110	0.42	**0** **.** **4** **5**	0.39	**0** **.** **4** **4**
T0953s2-D3	13	93	**0** **.** **3** **1**	**0** **.** **3** **1**	**0** **.** **2** **7**	**0** **.** **2** **7**
T0960-D2	13	84	0.37	**0** **.** **4** **2**	0.36	**0** **.** **3** **9**
T0898-D2	12	55	**0** **.** **4** **6**	0.44	**0** **.** **4** **5**	0.43
T0859-D1	12	129	0.29	**0** **.** **3** **2**	0.27	**0** **.** **3** **1**
T0897-D1	12	138	0.30	**0** **.** **3** **1**	0.26	**0** **.** **2** **8**
T0886-D1	12	69	0.47	**0** **.** **4** **9**	0.45	**0** **.** **4** **6**
T0953s1-D1	13	67	**0** **.** **5** **0**	0.46	**0** **.** **4** **8**	0.45

Highest values for each target are marked in bold

Table 8(d) shows the *p*-values obtained in 1-sided statistical significance tests that pitch Evo-Diverse against Rosetta, evaluating the null hypothesis that Evo-Diverse performs similarly or worse than Rosetta. Both Fisher’s and Barnard’s test are conducted, and *p*-values less than 0.05 (which reject the null hypothesis) are marked in bold. Table 8(d) shows that the null hypothesis is not rejected with 95% confidence and the performance improvement of Evo-Diverse over Rosetta is not statistically significant.

**Table 8:** *p*-values obtained by 1-sided Fisher’s and Barnard’s tests on the CASP dataset for head-to-head comparison of the algorithms on lowest energy and average energy of the best 10 decoys (a), lowest lRMSD and average lRMSD of the best 10 decoys (b), highest TM-score and average TM-score of the best 10 decoys (c), and highest GDT_TS score and average GDT_TS score of the best 10 decoys (d)

(a)	Test	Lowest energy	Avg. energy of the best 10
	Fisher’s	0.08945	**0.01151**
	Barnard’s	0.05789	**0.005909**
(b)	Test	Lowest lRMSD	Avg. lRMSD of the best 10
	Fisher’s	0.3281	**0.002739**
	Barnard’s	0.2617	**0.001288**
(c)	Test	Highest TM-score	Avg. TM-score of the best 10
	Fisher’s	0.6719	0.5
	Barnard’s	0.9991	0.4119
(d)	Test	Highest GDT_TS score	Avg. GDT_TS score of the best 10
	Fisher’s	0.5	0.5
	Barnard’s	0.4119	0.4119

All tests evaluate the null hypothesis that Evo-Diverse does *not* perform better than Rosetta, *p*-values less than 0.05 are marked in bold

## Conclusion

This paper presents a novel conformation sampling algorithm, Evo-Diverse, that operationalizes the multi-objective, stochastic optimization framework. The algorithm does not use total energy as a basis of selection but instead makes use of non-domination rank and crowding distance in its selection operator to encourage conformation diversity.

Yet, the results show that Evo-Diverse reaches regions of lower total energy in the energy landscape of the benchmark dataset used here for evaluation, showcasing its higher exploration capability over the Rosetta decoy generation protocol and other, state-of-the-art multi-objective EAs that use total energy as an additional optimization objective. In addition, Evo-Diverse is able to reach comparable or lower lRMSDs than Rosetta, thus constituting a clear advantage over the current state-of-the-art multi-objective EAs.

It is worth noting that Evo-Diverse does not make use of an archive of decoys ever sampled, unlike other multi-objective EAs that do so to update the Pareto metrics for use in the selection operator. Evo-Diverse uses only the current population and their offspring to perform selection, thus saving storage overhead.

The presented results constitute a promising research direction in improving decoy generation, and future work will consider additional optimization objectives and variants of improvement and selection operators to apportion a fixed computational budget. Of particular interest are directions of research that attenuate dependence on protein energy models and permit as optimization objectives learned rather than physics-based models of structural integrity and nativeness.

## Abbreviations

aa: Amino acid
EA: Evolutionary algorithm
lRMSD: Least root-mean-squared-deviation
PC: Pareto count
PDB: Protein data bank
PR: Pareto rank
PSP: Protein structure prediction

## Footnotes

1 The term conformation, though often interchanged with structure, refers to an assignment of values to variables selected to represent a spatial arrangement of the chain of amino acids. These variables can be Cartesian coordinates, angles, or others.

2 Work in [9] analyzes Rosetta energy/scoring functions and reports that, while these functions have improved, false minima are found on generated conformation/decoy datasets.

## References

[1] Blaby-Haas CE, de Crécy-Lagard V (2013). Mining high-throughput experimental data to link gene and function. Trends Biotechnol.

[2] Berman HM, Henrick K, Nakamura H (2003). Announcing the worldwide Protein Data Bank. Nat Struct Biol.

[3] Boehr DD (2008). Wright PE: How do proteins interact?. Science.

[4] Maximova T, Moffatt R, Ma B, Nussinov R, Shehu A (2016). Principles and Overview of Sampling Methods for Modeling Macromolecular Structure and Dynamics. PLoS Comp Biol.

[5] Kryshtafovych A, Barbato A, Fidelis K, Monastyrskyy B, Schwede T, Tramontano A (2014). Assessment of the assessment: evaluation of the model quality estimates in CASP10. Proteins.

[6] Leaver-Fay A, Tyka M, Lewis SM, Lange OF, Thompson J, Jacak R (2011). ROSETTA3: an object-oriented software suite for the simulation and design of macromolecules. Methods Enzymol.

[7] Xu D, Zhang Y (2012). Ab initio protein structure assembly using continuous structure fragments and optimized knowledge-based force field. Proteins Struct Funct Bioinf.

[8] Nussinov R, Wolynes PG (2014). A second molecular biology revolution? The energy landscapes of biomolecular function. Phys Chem Chem Phys.

[9] Rubenstein AB, Blacklock K, Nguyen H, Case DA, Khare SD. Systematic Comparison of Amber and Rosetta Energy Functions for Protein Structure Evaluation. J Chem Theory Comput. 2018;:6321–6322. [Preprint]. 10.1021/acs.jctc.8b00303 30240210

[10] Shehu A, Aluru S (2013). Probabilistic Search and Optimization for Protein Energy Landscapes. Handbook of Computational Molecular Biology.

[11] De Jong KA (2006). Evolutionary Computation: a Unified Approach.

[12] Zhang G, Ma L, Wang X, Zhou X. Secondary Structure and Contact Guided Differential Evolution for Protein Structure Prediction. IEEE/ACM Trans Comput Biol Bioinf. 2018;:1–1. ISSN=1545-5963, 10.1109/TCBB.2018.2873691. 10.1109/TCBB.2018.2873691 30295627

[13] Zhang GJ, Zhou GX, Yu XF, Hao H, Yu L (2017). Enhancing protein conformational space sampling using distance profile-guided differential evolution. IEEE/ACM Trans Comput Biol and Bioinf.

[14] Shehu A. A Review of Evolutionary Algorithms for Computing Functional Conformations of Protein Molecules In: Zhang W, editor. Computer-Aided Drug Discovery, Methods in Pharmacology and Toxicology. Springer Verlag: 2015.

[15] Olson B, De Jong KA, Shehu A (2013). Off-Lattice Protein Structure Prediction with Homologous Crossover. Conf on Genetic and Evolutionary Computation (GECCO).

[16] Olson B, Shehu A. Multi-Objective Stochastic Search for Sampling Local Minima in the Protein Energy Surface. In: ACM Conf on Bioinf and Comp Biol (BCB). Washington: 2013. p. 430–9.

[17] Olson B, Shehu A. Multi-Objective Optimization Techniques for Conformational Sampling in Template-Free Protein Structure Prediction. In: Intl Conf on Bioinf and Comp Biol (BICoB). Las Vegas: 2014. p. 143–8.

[18] Shmygelska A, Levitt M (2009). Generalized ensemble methods for de novo structure prediction. Proc Natl Acad Sci USA.

[19] Deb K, Agrawal S, Pratap A, Meyarivan T (2002). A fast and elitist multi-objective genetic algorithm: NSGA-II. IEEE Trans Evol Comput.

[20] Meiler J, Baker D (2003). Coupled prediction of protein secondary and tertiary structure. Proc Natl Acad Sci USA.

[21] DeBartolo J, Hocky G, Wilde M, Xu J, Freed KF, Sosnick TR (2010). Protein structure prediction enhanced with evolutionary diversity: SPEED. Protein Sci.

[22] Molloy K, Saleh S, Shehu A (2013). Probabilistic Search and Energy Guidance for Biased Decoy Sampling in Ab-initio Protein Structure Prediction. IEEE/ACM Trans Comput Biol and Bioinf.

[23] McLachlan AD (1972). A mathematical procedure for superimposing atomic coordinates of proteins. Acta Crystallogr A.

[24] Zhang Y, Skolnick J (2004). Scoring function for automated assessment of protein structure template quality. Proteins.

[25] Zemla A, Venclovas C, Moult J, Fidelis K (1999). Processing and analysis of CASP3 protein structure predictions. Proteins.

[26] Zemla A, Venclovas C, Moult J, Fidelis K (2001). Processing and evaluation of predictions in CASP4. Proteins.

[27] Fisher RA (1922). On the interpretation of *χ*^2^ from contingency tables, and the calculation of P. J Roy Stat Soc.

[28] Barnard GA (1945). A new test of 2x2 tables. Nature.

[29] McNicholas S, Potterton E, Wilson KS, Noble MEM (2011). Presenting your structures: the CCP4mg molecular-graphics software. Acta Cryst.

